# Soil pathogen-aphid interactions under differences in soil organic matter and mineral fertilizer

**DOI:** 10.1371/journal.pone.0179695

**Published:** 2017-08-17

**Authors:** Stijn van Gils, Giovanni Tamburini, Lorenzo Marini, Arjen Biere, Maaike van Agtmaal, Olaf Tyc, Martine Kos, David Kleijn, Wim H. van der Putten

**Affiliations:** 1 Department of Terrestrial Ecology, Netherlands Institute of Ecology (NIOO-KNAW), Wageningen, The Netherlands; 2 DAFNAE-Entomology, University of Padova, Padova, Italy; 3 Department of Microbial Ecology, Netherlands Institute of Ecology (NIOO-KNAW), Wageningen, The Netherlands; 4 Department of Life Sciences (Silwood Park), Imperial College London, London, United Kingdom; 5 Plant Ecology and Nature Conservation Group, Wageningen University and Research, Wageningen, The Netherlands; 6 Animal Ecology Team, Alterra – Wageningen University and Research, Wageningen, The Netherlands; 7 Resource Ecology Group, Wageningen University and Research, Wageningen, The Netherlands; 8 Laboratory of Nematology, Wageningen University and Research, Wageningen, The Netherlands; Institute for Sustainable Plant Protection, C.N.R., ITALY

## Abstract

There is increasing evidence showing that microbes can influence plant-insect interactions. In addition, various studies have shown that aboveground pathogens can alter the interactions between plants and insects. However, little is known about the role of soil-borne pathogens in plant-insect interactions. It is also not known how environmental conditions, that steer the performance of soil-borne pathogens, might influence these microbe-plant-insect interactions. Here, we studied effects of the soil-borne pathogen *Rhizoctonia solani* on aphids (*Sitobion avenae*) using wheat (*Triticum aestivum*) as a host.

In a greenhouse experiment, we tested how different levels of soil organic matter (SOM) and fertilizer addition influence the interactions between plants and aphids. To examine the influence of the existing soil microbiome on the pathogen effects, we used both unsterilized field soil and sterilized field soil.

In unsterilized soil with low SOM content, *R*. *solani* addition had a negative effect on aphid biomass, whereas it enhanced aphid biomass in soil with high SOM content. In sterilized soil, however, aphid biomass was enhanced by *R*. *solani* addition and by high SOM content. Plant biomass was enhanced by fertilizer addition, but only when SOM content was low, or in the absence of *R*. *solani*.

We conclude that belowground pathogens influence aphid performance and that the effect of soil pathogens on aphids can be more positive in the absence of a soil microbiome. This implies that experiments studying the effect of pathogens under sterile conditions might not represent realistic interactions. Moreover, pathogen-plant-aphid interactions can be more positive for aphids under high SOM conditions. We recommend that soil conditions should be taken into account in the study of microbe-plant-insect interactions.

## Introduction

The importance of aboveground-belowground invertebrate interactions in plant defence has been well acknowledged [1–4]. Belowground herbivory on plant roots may induce resistance or susceptibility in plant tissues to aboveground herbivores, as well as influence interactions with organisms at higher trophic levels [5]. Aboveground-aboveground interactions between microbes and insects mediated by the plant have also been well acknowledged [6]. However, only recently, interest in plant-mediated above-belowground interactions involving microbes and insects has emerged [7], mainly focusing on plant-mediated interactions between aboveground insects and belowground symbiotic mutualists, such as arbuscular mycorrhizal fungi [8–10] and plant growth promoting rhizobacteria [11, 12]. Few studies have considered interactions between belowground pathogenic microbes and aboveground herbivores [13]. Plant-microbe-insect interactions also depend on environmental conditions such as soil fertility [14], but these effects have not been well studied. Here we examine how soil organic matter (SOM) and mineral fertilizer influence interactions among a pathogenic soil fungus, a crop plant, and an aboveground aphid.

In natural ecosystems, as well as in agro-ecosystems, most plant species are attacked by numerous herbivores and pathogens that simultaneously subtract energy and nutrients from the plant [15]. Plants defend against enemies with an array of constitutive or induced, direct and indirect defences, including the induction of chemical defence compounds [16]. Plant defences against necrotrophic pathogens [17] and chewing insects are predominantly regulated via the jasmonic acid (JA) signal transduction pathway [18], whereas the salicylic acid (SA) pathway is triggered by biotrophic pathogenic fungi [19] and phloem feeding herbivores, such as aphids [20, 21]. The JA and SA pathways can interact via antagonistic crosstalk [22, 23], meaning that induction of JA signalling can lead to a decrease in SA mediated defences and vice versa [24]. It has therefore been suggested that infection with a necrotrophic pathogenic soil fungus such as *Rhizoctonia solani*, would most likely result in an increased performance of aphids such as *Sitobion avenae* [25]. However, there seems to be no general support for this suggestion [25], perhaps because pathogens may also affect plant nutrient uptake directly or via competition with other soil organisms leading to differences in plant primary and secondary chemical composition that are not related to the JA-SA crosstalk [26].

It has been well-established that aphids are affected by soil fertility, which influences plant growth and chemistry including secondary metabolites and, consequently, quantity and quality of food for aphids [27, 28]. Soil fertility can be enhanced by adding mineral fertilizer, but SOM also influences soil fertility. Effects of mineral fertilizer and SOM on plants may differ in various aspects, for example because of the rate at which nutrients become available [29]. Mineral fertilizers may be more quickly available than nutrients from SOM, which first need to be mineralized by the soil microbiome [30], resulting in a lower carbon to nitrogen (C:N) ratio of plants growing with mineral fertilizer. A low C:N ratio enhances the quality of phloem sap for phloem-feeding herbivores, such as aphids [31]. Variation in SOM content may likewise affect plant quality for aphids [32], however, considering the rate at which nutrients become available, SOM is expected to have weaker effects on the C:N ratio than mineral fertilizer supply. It is therefore expected that aphids will not respond as strongly to enhanced SOM content as to mineral fertilizer supply. Interestingly, experimental studies have provided mixed support for this expectation; some studies find support [33] whereas Garratt, Wright [34] found no significant overall effect in a meta-analysis.

Fertility management practices, including practices to enhance SOM content, can influence soil organisms including soil-borne pathogens such as *R*. *solani*, which have a weaker negative impact on plant biomass when plants are well supplied with nitrogen [35]. Subsequently, any change in the relative abundance of organisms in the soil microbiome may alter the systemic induction of plant defences [36] and, therefore, aphid performance. Also abiotic stress situations can alter microbe-plant-insect interactions [37]. Indeed, it has been shown that specific effects of mineral fertilizer on aphid performance largely depend on the composition of the soil community [38]. Also SOM- and fertilizer-induced changes in soil biota [39] may indirectly affect aphid performance by changing the magnitude or direction of plant-mediated interactions between soil-borne pathogens and aphids. This could be due either to shifts in the abundance of decomposer organisms that alter the nutritional status of plants, which may alter plant induced defence responses to soil-borne pathogens, or to shifts in the abundance of antagonists of the soil-borne pathogens [40].

In the present study we experimentally exposed spring wheat (*Triticum aestivum*) plants, an important crop species, to aphids (*S*. *avenae*), an important pest species, to two levels of SOM, two levels of mineral fertilizer, and with or without the addition of the soil borne fungal pathogen *R*. *solani*. We tested the following hypotheses: (1) Enhanced SOM content and higher mineral fertilizer supply decrease the effect of *R*. *solani* on aphid biomass and its negative effect on plant biomass. (2) Mineral fertilizer supply reduces plant C:N ratio more than an increase in SOM content, so that aphid biomass will be higher under enhanced mineral fertilizer supply than under enhanced SOM content. (3) Addition of *R*. *solani* affects aphid biomass strongest when the pathogen is inoculated to sterilized soils, which lack a soil microbiome that may control the fungus.

## Materials and methods

### Study design

We grew spring wheat (*Triticum aestivum*) under exposure to grain aphids (*Sitobion avenae*) in unsterilized and sterilized soil. Plants growing in unsterilized soil were exposed to all combinations of two levels of soil organic matter (SOM) content, high and low mineral fertilizer supply and presence or absence of the fungus *Rhizoctonia solani*. Each combination was replicated 14 times, bringing the total to 2×2×2×14 = 112 experimental units. Plants growing in sterilized soil were exposed to all combinations of two levels of SOM content and presence or absence of *R*. *solani* (i.e. we had no fertilization treatment, as sterilization already leads to a nutrient flush). Each combination was replicated 8 times, bringing the total to 2×2×8 = 32 units. All treatments from both sterilized and unsterilized soil were fully randomized in one greenhouse. We studied main and interaction effects on aphid performance (*S*. *avenae*) and the biomass and C:N ratio of its host plant spring wheat (*T*. *aestivum*).

### Treatments

We placed six pairs of seeds of *T*. *aestivum* (var. Tybalt) in four-litre pots (diameter ~20 cm) filled with soils consisting of 1.7% (Low) or 3.1% (High) SOM content. Soils were taken from a soil health experiment from a loamy fine sand area in the South East of the Netherlands (51°32'26.0"N; 5°51'13.0"E, see [41] for a full description of this experiment). All soils were collected at an experimental farm that is owned by Wageningen University & Research. The soil was collected with permission by employees from this university. To study the effect of SOM on aphid performance, independent of all other physical, chemical and biological properties of the soil that might affect yield and aphid performance, we obtained the two SOM treatments by mixing different proportions of two soil layers that differed in SOM but that originated from the same area. We used a concrete mixer for mixing the soils. The two soil layers were the top layer, 0–20 cm (3.4% SOM) and the C horizon (~100 cm depth) that hardly contained any SOM. The high SOM treatment was obtained by mixing the C horizon with the top layer in a 1:9 mixture, whereas the low SOM treatment was obtained using a mixture of 1:1. For the sterilized soil treatments we sterilized the soils twice during 20 minutes (40 minutes total) at 121°C using an autoclave.

After germination we removed the smaller of the two plants in a pair to get six plants per pot. One day after sowing, the high fertilizer supply treatment plants received 60% of the total fertilizer supplied during the experiment. The remaining 40% of fertilizer was provided at tiller development. In total we supplied 0.3 gram N.pot^-1^, which corresponds with an average nitrogen supply for spring wheat of around 130 kg N.ha^-1^. Nitrogen was supplied in a dissolved form of NO_3_^-^, (mainly Ca(NO_3_)_2_), enriched with half a litre ½ Hoagland solution that also contains other macro and micro nutrients (see [42] for composition of the solution). All other plants, including all plants growing in the sterilized soil, received fertilizer at the low supply rate. These plants received 10% of this solution completed with tap water to add equal volumes of water to every pot. In addition plants received ample water; on average 300 ml per pot per week.

Six days later, at tiller development, we inoculated half the pots with two 5 mm plugs of the fungus *R*. *solani* (AG-8) [43], which had grown for one week on Petri dishes with 1/5^th^ Potato Dextrose Agar (PDA; 29 gL^-1^ Oxoid CM 139). Pots were inoculated at rooting depth, around 4 cm depth, at both sides of the pot and plugs were always taken from two randomly selected agar plates to avoid any bias from a potential plate effect.

Three weeks after tiller development, each pot was covered by a gauze net (mesh size with openings of 150 μm diameter) and infested with six aphids per pot. The single adult apterous aphids were carefully placed with a fine brush on the biggest leaf of each of the six plants. Seven weeks after tiller development (hence four weeks after aphid infestation), aphids were carefully removed using a brush, put in a tube and weighted to obtain the aphid fresh weight (mg). Eight weeks after tiller development, plants were at that time at flowering stage, aboveground plant biomass—shoots and spikes—were harvested, oven dried at 70°C for 48 h and weighted. Subsequently we obtained the C:N ratio of the plant shoots, because most of the aphids fed on the shoots. To obtain C:N ratio, shoot plant material was homogenized and grinded to a fine powder and oven dried again for 24 h at 70°C to estimate carbon (C) and nitrogen (N) concentration. Prior to analysis tin cups were filled with 3–6 mg of sample powder and analysed using combustion-reduction with an element analyser (Thermo flash EA 1112, Thermo Fisher Scientific Inc., Waltham, USA).

### Analyses

To test hypotheses 1 and 2 we analysed data from the plants growing in unsterilized soil using a type 3 ANOVA. We ran separate models for the three response variables, aboveground plant biomass (shoots and spikes), fresh aphid biomass and plant quality (C:N ratio) and used SOM, fertilizer supply level and *R*. *solani* addition and all possible interactions as explanatory variables. We tested whether residuals followed a normal distribution, using Shapiro-Wilk tests. Residuals of the data on aphid biomass and C:N ratio were not normally distributed and were therefore √(ln+1) and √ transformed, respectively. We checked whether variances were equal using Bartlett's test. Afterwards, we ran a Tukey Honest Significant Difference (HSD) contrast test as a post hoc to see which combinations were significantly different from each other. Moreover, we looked how aboveground plant biomass, fresh aphid biomass, and C:N ratio were correlated to each, other using Pearson Correlations.

To test hypothesis 3, we combined the data from sterilized and unsterilized pots, the latter not receiving mineral fertilizer. Since sample sizes were not equal (i.e. we had fewer pots per combination for the sterilized soil), we performed this analysis using a linear model. Also here, we tested whether residuals followed a normal distribution, using Shapiro-Wilk tests, resulting in √(ln+1) transformation of the data.

All analyses were performed with R 3.2.2 (R Core Team).

## Results

### Effect of mineral fertilizer supply and SOM on plant- and aphid biomass and plant C:N ratio

**Fig 1:**
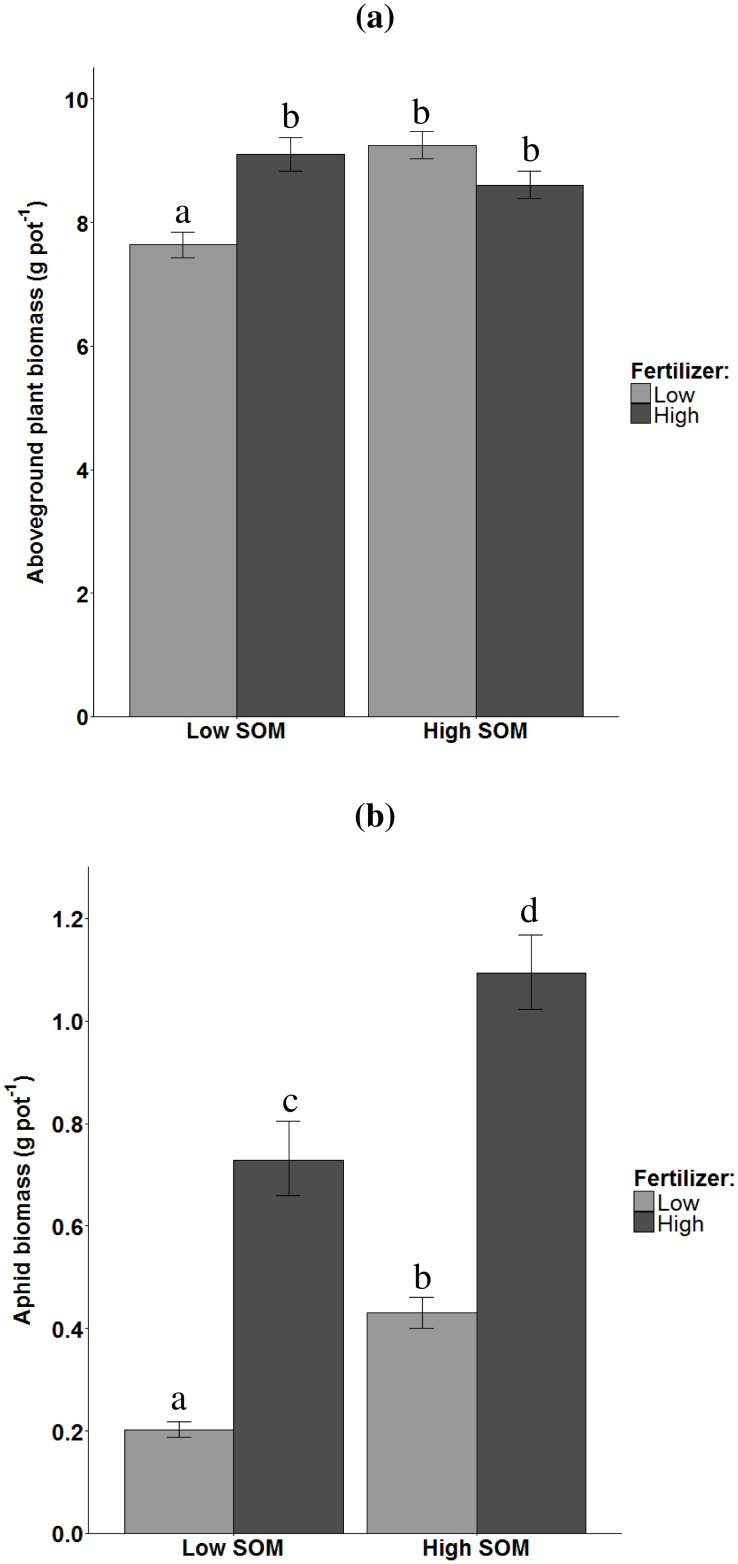
Effects of soil organic matter (SOM) content (low, high) and mineral fertilizer supply (low, high) on plant- and aphid biomass on unsterilized soil. a) Plant biomass of *Triticum aestivum*. b) Aphid biomass of *Sitobion avenae*. Error bars represent standard errors. Significant differences are indicated by different letters (Tukey Honest Significant Difference contrast test).

**Fig 2:**
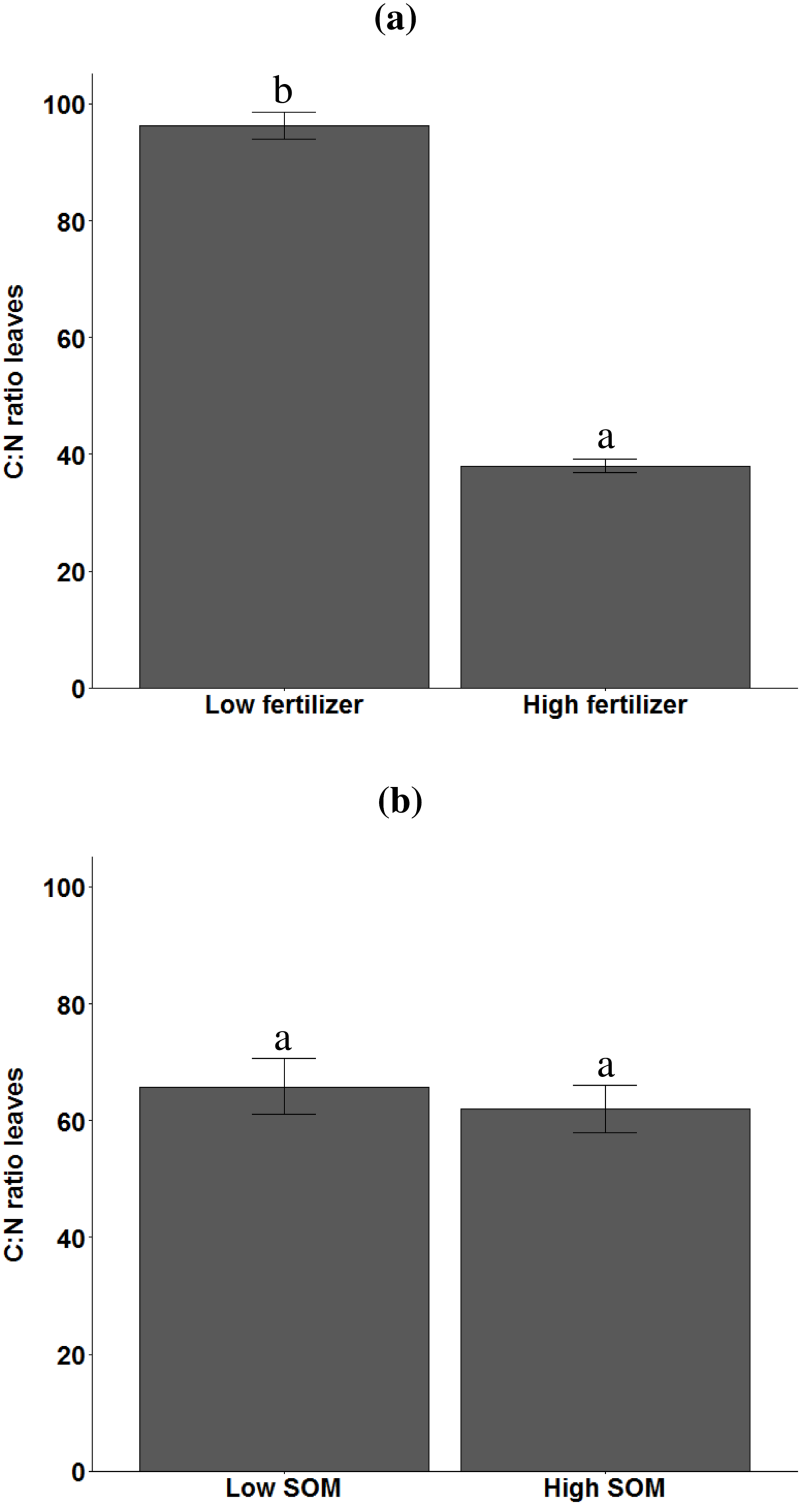
Effects of mineral fertilizer supply (low, high) and soil organic matter (SOM) content (low, high) on C:N ratio of *Triticum aestivum* leaves in unsterilized soil. a) C:N ratio explained by mineral fertilizer supply. b) C:N ratio explained by SOM content. Error bars represent standard errors. Significant differences are indicated by different letters (Tukey Honest Significant Difference contrast test).

Fertilizer supply had a positive effect on aboveground plant biomass, but only under low SOM conditions, whereas high SOM content had only a positive effect on plant biomass under low mineral fertilizer supply (Fig 1a). There was more aphid biomass on plants growing in soil with high SOM content and if more mineral fertilizer was supplied (Fig 1b). However, the effect of fertilizer supply changed with SOM content (Table 1); in soil with high SOM content the positive effect of fertilizer supply on aphid biomass was greater than in soil with the lower SOM content (Fig 1b). Fertilizer supply was the only factor influencing shoot C:N ratio (Table 1). Fertilizer supply reduced C:N ratio by 60% compared to unfertilized soil, whereas the SOM content and had no effect on leaf C:N ratio (Fig 2a and 2b respectively). Plant biomass was neither related to leaf C:N ratio (r = -0.01, P = 0.90), nor to aphid biomass (r = -0.00, P = 0.99), but leaf C:N ratio was negatively related to aphid biomass (r = 0.76, P<0.0001).

**Table 1:** ANOVA models explaining fresh aphid biomass (*Sitobion avenae*, √(ln+1) transformed), aboveground dried plant biomass (stem and flower biomass) of wheat (*Triticum aestivum*) and plant C:N ratio (√ transformed) by soil organic matter (SOM) content, fertilizer supply, *Rhizoctonia solani* addition and all possible interactions. All analyses were done on unsterilized soil. Significant (P<0.05) effects are highlighted in bold, N = 104.

	Plant biomass		Aphid biomass		C:N ratio plant	
	F	*P*	F	*P*	F	*P*
**Main effects**						
SOM	0.03	0.8674	1.09	0.2990	0.02	0.8747
Fertilizer	0.54	0.4626	**22.75**	**0.0000**	**142.94**	**0.0000**
*R*. *solani*	**4.91**	**0.0290**	**4.82**	**0.0305**	0.44	0.5067
**Interactions**						
SOM:Fertilizer	**5.06**	**0.0268**	**3.96**	**0.0495**	0.00	0.9768
SOM:*R*. *solani*	3.26	0.0743	**7.49**	**0.0074**	0.21	0.6497
Fertilizer:*R*.*solani*	**8.40**	**0.0047**	3.75	0.0559	0.31	0.5786
SOM:Fertilizer:*R*.*solani*	1.64	0.2038	2.72	0.1021	2.50	0.1172

### Effect of adding *R*. *solani* on plant- and aphid biomass under different soil conditions

**Fig 3:**
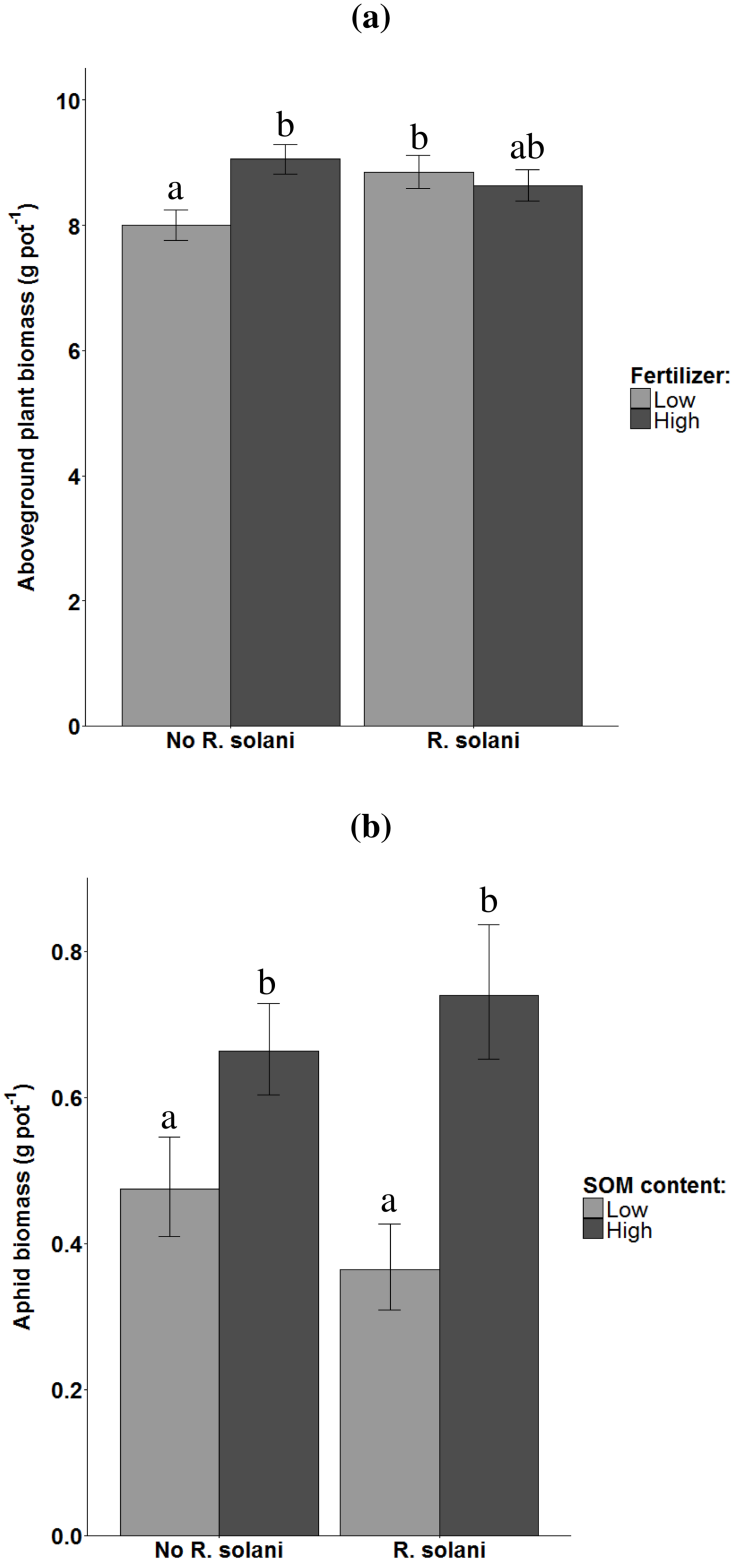
Effects of *Rhizoctonia solani* addition on plant- and aphid biomass under different soil conditions on unsterilized soil. a) Aboveground plant biomass of *Triticum aestivum* explained by *R*. *solani* addition and mineral fertilizer supply (low, high). b) Aphid biomass of *Sitobion avenae* explained by *R*. *solani* addition and soil organic matter (SOM) content (low, high). Error bars represent standard errors. Significant differences are indicated by different letters (Tukey Honest Significant Difference contrast test).

The addition of *Rhizoctonia solani* affected both plant and aphid biomass in unsterilized soils, but the effects were different depending on the soil conditions soil organic matter (SOM) content and fertilizer supply (Table 1). In soil with low mineral fertilizer supply, *R*. *solani* did not affect plant biomass, whereas under fertilized conditions *R*. *solani* cancelled the positive effect of mineral fertilizer supply on yield (Table 1, Fig 3a). The effect of adding *R*. *solani* on aphid biomass, depended on SOM content (Table 1). In soils with low SOM content *R*. *solani* addition tended to decrease aphid biomass, whereas it tended to increase aphid biomass in soil with high SOM content (Fig 3b), resulting in a significant interaction between SOM and *R*. *solani* addition (Table 1). The combination of *R*. *solani* addition and mineral fertilizer supply did not result in a significant interaction, although the trend was similar to the interaction between SOM content and *R*. *solani* addition (Table 1).

### Effects of R. solani under sterilized and unsterilized soil conditions

**Fig 4:**
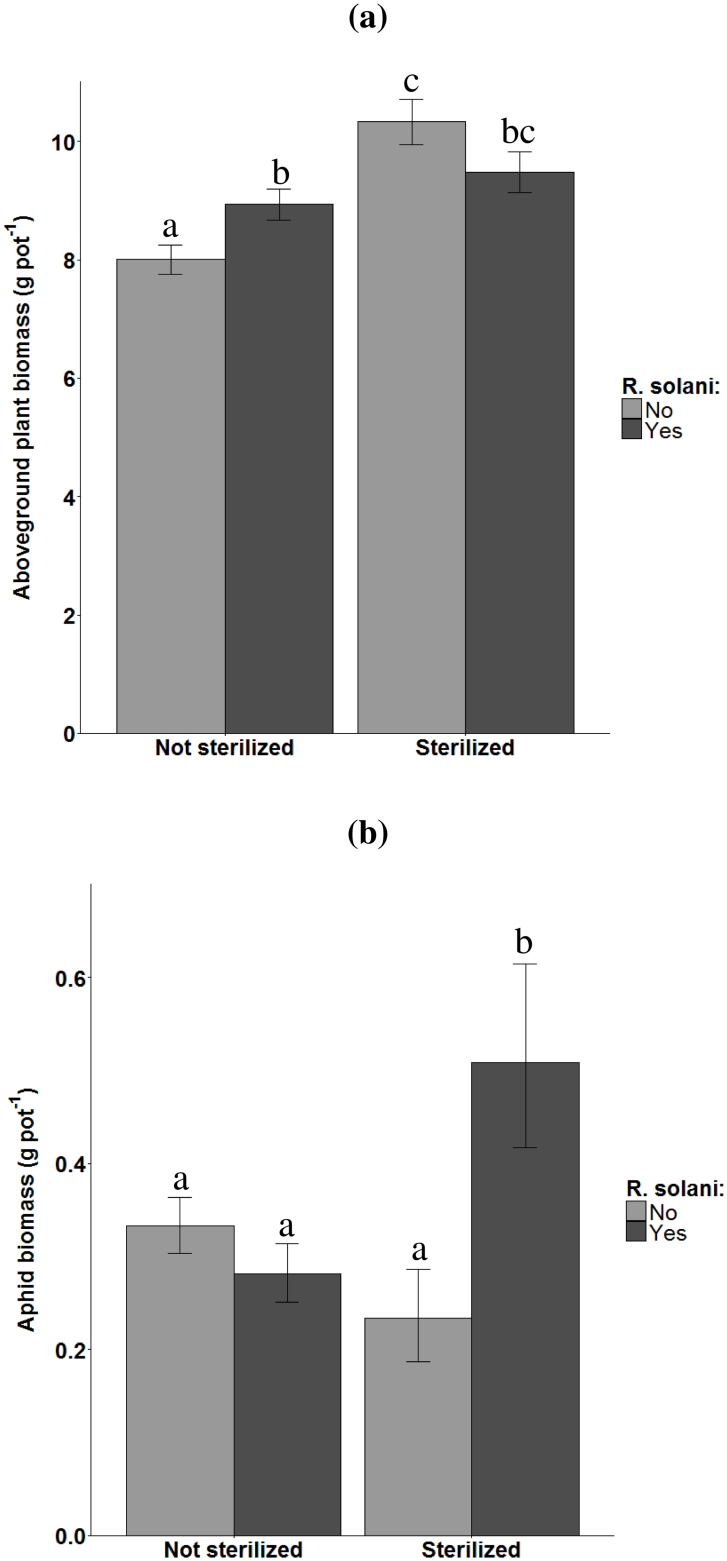
Effects of soil sterilization and *Rhizoctonia solani* addition on aboveground plant biomass of and fresh aphid biomass. a) Effects on aboveground plant biomass of *Triticum aestivum*. b) Effects on fresh aphid biomass of *Sitobion avenae*. Error bars represent standard errors. Significant differences are indicated by different letters (Tukey Honest Significant Difference contrast test).

The effect of *R*. *solani* addition on plant biomass changed with soil sterilization (F = 6.97, P = 0.01, N = 88). In non-sterilized soil, *R*. *solani* addition increased plant biomass, whereas it tended to decrease plant biomass under sterilized conditions (Fig 4a). The effects of *R*. *solani* infection on aphid biomass depended on soil sterilization (interaction: t = 4.20, P = 0.0001, N = 88, Fig 4b). In unsterilized soil, addition of *R*. *solani* had no effect on aphid biomass, but in sterilized soil, addition of *R*. *solani* had a substantial positive effect on aphid biomass (Fig 4b). The effects of sterilization, SOM content and *R*. *solani* addition on biomass of aphids and plants and C:N ratio of leaves are presented in S1 Appendix.

## Discussion

Various studies have shown that belowground organisms, such as insects [44], nematodes [45], and mycorrhizal fungi [9, 46, 47] can alter the performance of aboveground invertebrate organisms. It is also known that aboveground pathogens may alter the performance of aboveground herbivores [25]. However, as the effects of belowground pathogens on aboveground herbivores are not well studied [13], it remains unclear whether interactions between pathogenic microbes plants and insects are local or systemic [48]. Here, we demonstrate that a belowground pathogen may also influence aboveground plant-insect interactions and we show that effects depend on soil organic matter (SOM) content and mineral fertilizer supply.

We hypothesized that *Rhizoctonia solani* addition would lead to a decrease in plant biomass and an increase in aphid biomass and that enhanced SOM content and mineral fertilizer supply would reduce these effects. However, we found a pattern that opposed our hypothesis: under low SOM content *R*. *solani* addition tended to decrease aphid (*Sitobion avenae*) biomass, whereas it tended to increase aphid biomass under high SOM content. Supply of mineral fertilizer had a similar interaction effect with *R*. *solani* on aphid biomass, but this interaction was only marginally significant. Also in aboveground plant biomass we found a pattern opposing our hypothesis. As *R*. *solani* is known to have less negative effects on wheat growth under higher nitrogen conditions [35], we expected that SOM content or mineral fertilizer supply would negatively affect *R*. *solani* performance, leading to a less positive effect of *R*. *solani* on aphid performance. We observed, however, that *R*. *solani* addition led to an increase in aboveground plant biomass when the low fertilizer treatment was supplied and that there was no effect of aphids when we supplied the full mineral fertilizer treatment. This result was not expected as *R*. *solani* AG-8 is known to be pathogenic to wheat *Triticum aestivum* [49]. Potentially, an *R*. *solani* infection increases immunity of the crop plant against aphids in some situations.

Our second hypothesis was that mineral fertilizer supply would reduce plant C:N ratio more than an increase in SOM content, so that aphid biomass will be higher under enhanced mineral fertilizer supply than under enhanced SOM content. We expected that high SOM content would have a smaller effect on C:N ratio than mineral fertilizer supply [31], leading to relatively weaker performance of aphids. Indeed, our data showed that mineral fertilizer supply strongly decreased plant C:N ratio. High SOM content, however, increased plant biomass without affecting plant C:N ratio when no mineral fertilizer was supplied. These different responses of plant biomass production and C:N ratio to SOM and fertilizer could explain why an increase in SOM content could have a smaller positive effect on aphid biomass than mineral fertilizer supply, while the effect on plant biomass is similar. This could be explained by a competition for nutrients among micro-organisms that decompose SOM and the plant [33]. However, *S*. *avenae* showed still an increase in biomass under higher SOM content. Possibly, *S*. *avenae* does not solely respond to the decrease in C:N ratio after fertilization per se. Instead, it might respond to extra increase in plant biomass after mineral fertilizer supply, potentially explaining why *S*. *avenae* responds after fertilization in some studies [50], but not in others [51].

We expected that addition of *R*. *solani* affected aphid biomass strongest when the pathogen is inoculated to sterilized soils, as these sterilized soils lack microbiome components that may control the fungus [40]. In support of this hypothesis we found that aphid biomass increased when *R*. *solani* was added to sterilized soil. The positive effect of *R*. *solani* on aphid performance is also in line with predictions from defence signalling interactions (Lazebnik et al. 2014). Necrotrophic fungi, such as *R*. *solani* trigger defence responses mediated by the jasmonic acid (JA) signalling pathway, which through JA-SA crosstalk can lead to a suppression of salicylic acid (SA) mediated defence [24]. However, we did not measure hormone levels in plants, so that this possible mechanistic explanation needs further study.

## Conclusions

We conclude that the outcome of a soil pathogen-aphid interaction may depend on the SOM content as a higher SOM content leads to a more positive effect of *R*. *solani* on aphids. We therefore recommend that soil conditions, such as SOM content, should be taken into account in the study of microbe-plant-insect interactions. We also show that belowground pathogens influence aphid performance and that this effect depends on the presence of a soil microbiome. We saw that, under the absence of a soil microbiome, the effect of *R*. *solani* on aphid biomass became more positive, implying that experiments studying the effect of soil pathogens under sterile conditions might not represent realistic outcomes of interactions. Further studies are needed to further unravel mechanisms at the plant hormone level, and to test how this knowledge can be used to understand plant exposure to combinations of belowground and aboveground natural enemies at field scale, for instance in agricultural systems.

## Supporting information

S1 Appendix Fresh aphid biomass, aboveground plant biomass, and C:N ratio explained by soil sterilization, soil organic matter content and *Rhizoctonia solani* addition. (DOC)

S1 Fig Effects of soil organic matter (SOM) content (low, high) on fresh aphid biomass in both sterilized and unsterilized soil. (DOC)

S2 Fig Effects of soil organic matter (SOM) content (low, high) on aboveground plant biomass in both sterilized and unsterilized soil. (DOC)

S3 Fig Effects of soil sterilization, soil organic matter (SOM) content (low, high) and *Rhizoctonia solani* addition on C:N ratio of *Triticum aestivum* leaves. (DOC)

S1 Table Linear models explaining fresh aphid biomass (*Sitobion avenae*, √(ln+1) transformed), aboveground dried plant biomass (stem and flower biomass) of wheat (*Triticum aestivum*) and plant C:N ratio (√ transformed) by soil sterilization, soil organic matter (SOM) content, *Rhizoctonia solani* addition and all possible interactions. (DOC)
