# Supplementary material for: Soil pathogen-aphid interactions under differences in soil organic matter and mineral fertilizer
Source: PLoS One. 2017 Aug 17;12(8):e0179695. doi: 10.1371/journal.pone.0179695 (PMC5560682; doi:10.1371/journal.pone.0179695)
Supplement: S1 Appendix — (DOC) [file pone.0179695.s001.doc]

**S1 Appendix. Fresh aphid biomass, aboveground plant biomass, and C:N ratio explained by soil sterilization, soil organic matter content and Rhizoctonia solani addition**

*Supporting Information to: Soil pathogen-aphid interactions under differences in soil organic matter and mineral fertilizer*

*Stijn van Gils, Giovanni Tamburini, Lorenzo Marini, Arjen Biere, Maaike van Agtmaal, Olaf Tyc, Martine Kos, David Kleijn, and Wim H. van der Putten.*

We tested whether soil sterilization, soil organic matter (SOM) content, Rhizoctonia solani addition and interactions among these factors affected fresh aphid biomass, aboveground dried plant biomass and C:N ratio of the plant leaves using linear models and a ge.

Fresh aphid biomass, aboveground plant biomass, and C:N ratio explained by soil sterilization, soil organic matter content and *Rhizoctonia solani* addition: We tested whether soil sterilization, soil organic matter (SOM) content, *Rhizoctonia solani* addition and interactions among these factors affected fresh aphid biomass, aboveground dried plant biomass and C:N ratio of the plant leaves using linear models. Fresh aphid biomass was affected by SOM content and the interaction between soil sterilization and *R. solani* addition (see main text and Table S1). Under high SOM content aphid biomass was higher (Fig S1). Plant biomass was affected by soil sterilization, SOM content and the interaction between soil sterilization and *R. solani* addition (main text and Table S1). Likewise aphid biomass, plant biomass was higher under high SOM content (Fig S2). The C:N ratio of the leaves was influenced by soil sterilization, SOM content, the interaction between soil sterilization and SOM content and the interaction between soil sterilization and *R. solani* addition. In sterilized soil, a higher SOM content let to a decrease in C:N ratio, whereas a higher SOM content did not let to a decrease in C:N ratio under unsterilized conditions (Fig S3a). In sterilized soils *R. solani* addition tended to increase C:N ratio, whereas it tended to decrease C:N ratio sterilized soils (Fig S3b).
