# Supplementary material for: Soil pathogen-aphid interactions under differences in soil organic matter and mineral fertilizer
Source: PLoS One. 2017 Aug 17;12(8):e0179695. doi: 10.1371/journal.pone.0179695 (PMC5560682; doi:10.1371/journal.pone.0179695)
Supplement: S2 Fig — (DOC) [file pone.0179695.s003.doc]

**Fig S2**

**Fig S2. Effects of soil organic matter (SOM) content (low, high) on aboveground plant biomass in both sterilized and unsterilized soil.** Significant differences are indicated by different letters (Tukey Honest Significant Difference contrast test).
