# Supplementary material for: Soil pathogen-aphid interactions under differences in soil organic matter and mineral fertilizer
Source: PLoS One. 2017 Aug 17;12(8):e0179695. doi: 10.1371/journal.pone.0179695 (PMC5560682; doi:10.1371/journal.pone.0179695)
Supplement: S3 Fig — (DOC) [file pone.0179695.s004.doc]

**Fig. S3**

**Fig S3. Effects of soil sterilization, soil organic matter (SOM) content (low, high) and *Rhizoctonia solani* addition on C:N ratio of *Triticum aestivum* leaves.** a) C:N ratio explained by soil sterilization and SOM content. b) C:N ratio explained by soil sterilization and *R. solani* addition. Error bars represent standard errors. Significant differences are indicated by different letters (Tukey Honest Significant Difference contrast test).
