# Supplementary material for: Soil pathogen-aphid interactions under differences in soil organic matter and mineral fertilizer
Source: PLoS One. 2017 Aug 17;12(8):e0179695. doi: 10.1371/journal.pone.0179695 (PMC5560682; doi:10.1371/journal.pone.0179695)
Supplement: S1 Table — (DOC) [file pone.0179695.s005.doc]

**Table S1. Generalized least squares model explaining fresh aphid biomass (*Sitobion avenae*, √(ln+1) transformed) and linear models explaining aboveground dried plant biomass (stem and flower biomass) of wheat (*Triticum aestivum*) and plant C:N ratio (√ transformed) by soil sterilization, soil organic matter (SOM) content, *Rhizoctonia solani* addition and all possible interactions. Significant (P<0.05) effects are highlighted in bold, N=88.**

|  | **Aphid biomass** | | **Plant biomass** | | **C:N ratio plant** | |
| --- | --- | --- | --- | --- | --- | --- |
|  | t | P | F | P | F | P |
| **Main effects** |  |  |  |  |  |  |
| Sterilization | -1.76 | 0.0821 | **33.65** | **<0.0001** | **44.29** | **<0.0001** |
| SOM | **-3.80** | **0.0003** | **28.69** | **<0.0001** | **20.91** | **<0.0001** |
| *R. solani* | -0.70 | 0.4859 | 1.73 | 0.1919 | 0.17 | 0.6829 |
| **Interactions** |  |  |  |  |  |  |
| Sterilization:SOM | 0.30 | 0.7673 | 1.53 | 0.2196 | **5.52** | **0.0212** |
| Sterilization:*R. solani* | **4.20** | **0.0001** | **6.97** | **0.0100** | **4.20** | **0.0436** |
| SOM:*R. solani* | -0.32 | 0.7508 | 0.16 | 0.6882 | 1.19 | 0.2780 |
| Sterilization:SOM:*R. solani* | 1.45 | 0.1520 | 0.01 | 0.9120 | 1.61 | 0.2077 |
